# Somatic mutations in a multigene panel and impact on prognosis based on TP53 status in Chinese HER2‐positive patients undergoing neoadjuvant therapy: A single‐institution retrospective cohort

**DOI:** 10.1002/cam4.6955

**Published:** 2024-02-01

**Authors:** Min Xiong, Xuliren Wang, Douwaner Liu, Bingqiu Xiu, Qi Zhang, Weiru Chi, Chih Wan Goh, Liyi Zhang, Ming Chen, Hengyu Ren, Zhi‐Ming Shao, Benlong Yang, Jiong Wu

**Affiliations:** ^1^ Department of Breast Surgery, Key Laboratory of Breast Cancer in Shanghai Fudan University Shanghai Cancer Center Shanghai China; ^2^ Collaborative Innovation Center for Cancer Medicine Shanghai China

**Keywords:** HER2‐positive breast cancer, human epidermal growth factor receptor 2 (HER2), neoadjuvant therapy (NAT), pathological complete response (pCR), somatic mutation

## Abstract

**Background:**

Gene mutations play a crucial role in the occurrence and development of tumors, particularly in breast cancer (BC). Neoadjuvant therapy (NAT) has shown greater clinical benefit in HER2‐positive breast cancer. However, further clinical investigation is needed to fully understand the correlation between genetic mutations and NAT efficacy and the long‐term prognosis in HER2‐positive BC.

**Methods:**

This was a retrospective cohort study of 222 patients receiving NAT between 2017 and 2021 in the Department of Breast Surgery of Fudan University Shanghai Cancer Center. Tumor samples from these patients were subjected to Next Generation Sequencing (NGS) to analyze mutations in 513 cancer‐related genes. This study aimed to investigate the association between these genetic mutations and postoperative pathological complete response (pCR), as well as their impact on disease‐free survival (DFS).

**Results:**

In total, 48.65% patients reached pCR, ER‐negative status (*p* < 0.001), PR‐negative status (*p* < 0.001), Ki67 ≥ 20 (*p* = 0.011), and dual‐targeted therapy (*p* < 0.001) were all associated with enhanced pCR rates. The frequency of somatic alterations in TP53 (60%), PIK3CA (15%), and ERBB2 (11%) was highest. In the HER2+/HR‐ cohort, patients who achieved pCR had a significant benefit in prognosis (HR = 3.049, *p* = 0.0498). KMT2C (*p* = 0.036) and TP53 (*p* = 0.037) mutations were significantly increased in patients with DFS events. Moreover, TP53 mutations had prognostic significance in HER2‐positive BC patients with HR‐negative (HR = 3.712, *p* = 0.027) and pCR (HR = 6.253, *p* = 0.027) status and who received herceptin‐only targeted therapy (HR = 4.145, *p* = 0.011).

**Conclusions:**

The genetic mutation profiles of Chinese HER2+ patients who received NAT were discrepant with respect to HR status or DFS events. TP53 mutations have significant prognostic value in patients with NAT for HER2‐positive BC and patients benefit differently depending on HR status, the neoadjuvant regimen and response, which highlights the significance of genetic factors in treatment customization based on individual genetic and clinical characteristics.

## Graphical abstract

This is the largest study to date addressing the relevance of diverse genotype mutations in Chinese HER2‐positive BC patients. TP53, PIK3CA, and ERBB2 showed the highest frequency of somatic alterations. The genetic mutation profiles of Chineses HER2‐positive patients who received NAT were discrepant with respect to HR status or DFS events. Somatic alterations in TP53 were related to prognosis and it benefited differently depending on HR status, the neoadjuvant regimen, and response. Our work hints at the high significance of a more precise formulation of molecular and molecular and genetic mutation subtypes for BC patients.

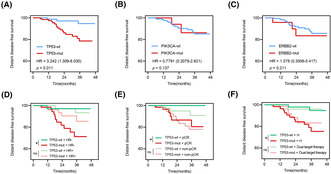

## 1 INTRODUCTION

Breast cancer (BC) is one of the most commonly diagnosed malignant tumors and is the leading cause of cancer death among females worldwide.^1^ BC is a heterogeneous disease at the molecular level. Molecular features include activation of human epidermal growth factor receptor 2 (HER2, encoded by ERBB2), hormone receptors (HRs, estrogen receptor and progesterone receptor), and diverse genetic mutationss.^2^ HER2 gene amplification or protein overexpression accounts for approximately 15%–20% of invasive breast cancer cases, which is an indicator of poor prognosis of BC and can be further typed.^3^

In recent years, an increasing number of clinical studies have focused on targeted therapies. Neoadjuvant therapy (NAT) has become a standard clinical practice used to downsize the tumor and increase the breast‐conserving surgery rate.^4^ The addition of herceptin on top of chemotherapy increased the pathological complete response (pCR) rate to 38%,^5^ and after intervention with both herceptin and pertuzumab, the pCR rate increased to approximately 70%.^6^ However, 30%–60% of patients with early HER2‐positive BC cannot achieve pCR after NAT. More biomarkers to predict efficacy and more therapeutic targets are needed.

BC has a variety of distinct molecular markers, gene expression profiles, and subtype‐specific patterns of genomic alterations that provide treatment guidance.^7^ The recent success of poly‐ADP ribose polymerase inhibitors (PARPi) in selectively treating breast cancers with germline BRCA1/2 mutations has proven the potential therapeutic efficiency in targeting genomic profiles.^8^ In addition, TP53, PIK3CA, and other genetic mutations were reported to affect the efficacy of targeted therapy and prognosis of patients with HER2‐positive BC.^9^ It has been reported that HER2‐positive breast cancers with activating mutations in PIK3CA are less likely to benefit from NAT.^10^, ^11^ Chemotherapy and radiotherapy cause DNA damage in tumor cells, and TP53 induces apoptosis after DNA damage.^12^ Many studies have proven that TP53 mutation can increase the pCR rate of neoadjuvant chemotherapy.^13^, ^14^

However, the effect of gene mutations on the response to HER2‐targeted NAT remains questionable. More importantly, there are several limitations in current research on genetic mutations, including inaccurate detection methods, insufficient samples, and inconsistent treatment plans.^15^ The study of molecular and genomic composition and mutational landscapes has reshaped our knowledge of identified subgroups of BC with diverse molecular etiologies and pathologies.^16^ Therefore, our research aimed to develop a deeper exploration of genomic alterations in HER2‐positive patients undergoing NAT and determine whether genetic mutations can be used as biomarkers to predict the efficacy of neoadjuvant therapy, exploring the relationship between genetic mutations and patient prognosis to the greatest extent among HER2‐positive BC patients.

## 2 MATERIALS AND METHODS

### 2.1 Patient cohort

The retrospective study focused on patients who underwent neoadjuvant therapy and genetic mutation testing. Immunohistochemistry and fluorescence in situ hybridization (FISH) detection were conducted on core needle biopsy puncture tissues; patients with HER2 3+ or HER2 2+/FISH + findings were included, while those with metastatic BC at the initial treatment were excluded. Study protocols were approved by the Ethical Review Community of Fudan University Shanghai Cancer Center (FUSCC), and all patients signed informed consent forms in accordance with institutional guidelines.

### 2.2 Study end point

Patients received neoadjuvant‐targeted therapy plus chemotherapy. Targeted therapy consisted of either herceptin‐only or a dual‐targeted approach, and surgery was performed after 6–8 cycles of neoadjuvant treatment. The primary end point in our study was pathological complete response (pCR), which was defined as the absence of invasive residual carcinoma in the breast and ipsilateral axillary lymph nodes (ypT0/is ypN0). The secondary end point was disease‐free survival (DFS), which was calculated from the date of surgery, and those alive without recurrence were censored at their last status date.^17^

### 2.3 FUSCC multigene panel of BC

The custom‐designed multigene panel comprised 513 genes that are closely related to the occurrence and development of BC based on the TCGA database, METABRIC database, MSKCC‐IMPACT database, WGS database of 560 cases of BC in the United States, and database of the Fudan University Cancer Institute. Data were collected using Illumina Real Time Analysis and assembled to fastq files using Illumina Bcl2Fastq2.^18^ Conditions for identifying a positive mutant gene included (1) variant allele frequency (VAF) ≥ 10% and (2) sequencing depth in the region ≥8.^19^ Mutation types included missense mutation, in frame deletion, nonstop mutation, frame shift insertion, splice site mutation, frame shift deletion, and multi hit.

### 2.4 Statistical analysis

The general characteristics data in this study were presented as the mean ± standard deviation (SD) for continuous variables and the number (percentage) for categorical variables, and the analysis of clinical information was completed by SPSS 20.0. The statistical methods included the chi‐square test, Fisher's exact test, and Kaplan–Meier survival analysis. For all analyses, bilateral *p* < 0.05 was considered significant. The “MutSigCV” algorithm was applied to screen 20 oncogenes with higher mutation frequencies, and the genetic mutations waterfall map was completed by the “maftools” package using R software and its packages (version 4.1.2).

## 3 RESULTS

### 3.1 Risk factors affecting the efficacy of NAT

In total, 222 HER2‐positive Chinese BC patients receiving NAT with no baseline metastasis between September 2017 and December 2021 in the FUSCC were included in this retrospective study. All of them were followed up continuously with a median follow‐up time of 23.8 months. The baseline clinical and pathological characteristics of the patient cohort are presented in Table 1. Of these, 108 (48.65%) patients reached pCR after NAT. The age of all patients ranged from 25 to 74 years, and the median age was 50 years. In total, 106 (47.75%) patients were ER‐positive, 62 (27.93%) patients were PR‐positive, and 208 (93.69%) patients had a Ki‐67 level ≥20%. Overall, 209 (94.14%) patients were scored as HER2 status 3+ through IHC, and 200 (90.09%) patients were HER2‐positive, as indicated by FISH. The majority of cases were at stages II‐III (II stage *n* = 116, 52.25%; III stage *n* = 106, 47.75%). Most patients were treated with H (Herceptin, 53.60%) or HP (Herceptin plus Pertuzumab, 29.28%). In addition, 12 (5.41%) patients quit during NAT treatment. In total, 200 (90.09%) patients received mastectomy, and 187 (84.23%) patients received axillary lymph node dissection (ALND). During the follow‐up period, 19 (8.56%) patients experienced DFS events. Seven patients had brain metastases, which were for the most frequent type of distant metastasis (Table S1).

**TABLE 1:** Baseline clinicopathological characteristics of the patients.

Patient characteristics	Number of patients (*N* = 222)	Percentage (%)
Year of diagnosis		
2017–2018	120	54.05
2019–2021	102	45.95
Age at diagnosis, years		
25–35	14	6.31
35–45	45	20.27
45–55	94	42.34
55–75	69	31.08
BMI		
<18.5	7	3.15
18.5 ≤ BMI < 24.0	125	56.31
24.0 ≤ BMI < 28.0	73	32.88
≥28.0	17	7.66
Family history of breast cancer		
No	206	92.79
Yes	16	7.21
Family history of other cancers		
No	170	76.58
Yes	52	23.42
cT (Pretreatment)		
T1	21	9.46
T2	127	57.21
T3	38	17.12
T4	36	16.22
cN (Pretreatment)		
N0	28	12.61
N1	136	61.26
N2	32	14.41
N3	26	11.71
Clinical stage (Pretreatment)		
II	116	52.25
III	106	47.75
pT (Postoperation)		
T0	108	48.65
T1	79	35.59
T2	14	6.31
T3	8	3.60
pN (Postoperation)		
N0	168	75.68
N1	41	18.47
N2	7	3.15
N3	6	2.70
Pathological stage (Postoperation)		
0	110	49.55
I	50	22.52
II	47	21.17
III	15	6.76
Miller–Payne Grade		
1	4	1.80
2	12	5.41
3	26	11.71
4	53	23.87
5	117	52.70
Unknown	10	4.50
ER status		
Negative	116	52.25
Positive	106	47.75
PR status		
Negative	160	72.07
Positive	62	27.93
Ki‐67		
<20%	14	6.31
≥20%	208	93.69
HER2 status IHC score		
2+	13	5.86
3+	209	94.14
FISH		
Negative	2	0.90
Positive	200	90.09
Unknown	20	9.01
Molecular subtype		
HER2+/HR‐	114	51.35
HER2+/HR+	108	48.65
Neoadjuvant‐targeted therapy		
H	119	53.60
HP	65	29.28
H + Pyrotinib	37	16.67
HL	1	0.45
Enough cycle		
Yes	210	94.59
No	12	5.41
Local therapy		
Breast‐conserving surgery	22	9.91
Mastectomy	200	90.09
Axillary surgery		
SLNB	35	15.77
ALND	187	84.23
pCR		
Yes	108	48.65
No	114	51.35
DFS events		
Yes	19	8.56
No	203	91.44

Subsequently, we conducted an investigation to identify the factors that influence the rate of pCR in patients. As neoadjuvant therapy continues to improve, there has been a rise in the utilization of dual‐targeted therapy. Our analysis, as presented in Table 2, revealed that dual‐targeted therapy exhibited significantly higher pCR rates compared to herceptin monotherapy (*p* < 0.001). Furthermore, the pCR rate for breast cancer cases diagnosed between 2019 and 2021 demonstrated a greater magnitude compared to those observed during 2017–2018 (*p* < 0.001). Patients with ER‐negative (*p* < 0.001) and PR‐negative (*p* < 0.001) status also exhibited improved pCR rates. Additionally, elevated Ki67 levels were found to be associated with an increased pCR rate (*p* = 0.011). Consequently, the primary risk factors that influenced pCR rates in our patient cohort were hormone receptor status and the regimen of neoadjuvant therapy.

**TABLE 2:** Risk factors affecting the efficacy of NAT.

Patient characteristics	non‐pCR		pCR		*p*
	Number of patients (*N* = 114)	Percentage (%)	Number of patients (*N* = 108)	Percentage (%)	
Year of diagnosis					
2017–2018	76	66.67	44	40.74	**<0.001**
2019–2021	38	33.33	64	59.26	
Age at diagnosis, years					
25–35	8	7.02	6	5.56	0.314
35–45	28	24.56	17	15.74	
45–55	47	41.23	47	43.52	
55–75	31	27.19	38	35.19	
BMI					
<18.5	7	6.14	5	4.63	0.443
18.5 ≤ BMI < 24.0	125	109.65	57	52.78	
24.0 ≤ BMI < 28.0	73	64.04	36	33.33	
≥28.0	17	14.91	10	9.26	
Family history of breast cancer					
No	109	95.61	97	89.81	0.121
Yes	5	4.39	11	10.19	
Family history of other cancers					
No	90	78.95	80	74.07	0.43
Yes	24	21.05	28	25.93	
cT (Pretreatment)					
T1	6	5.26	15	13.89	0.112
T2	67	58.77	60	55.56	
T3	19	16.67	19	17.59	
T4	22	19.3	14	12.96	
cN (Pretreatment)					
N0	14	12.28	14	12.96	0.165
N1	67	58.77	69	63.89	
N2	22	19.3	10	9.26	
N3	11	9.65	15	13.89	
Clinical stage (Pretreatment)					
II	57	50	59	54.63	0.505
III	57	50	49	45.37	
C2 therapeutic effect					
PD	1	0.88	0	0	**<0.001**
PR	57	50	77	71.3	
SD	13	11.4	1	0.93	
Unknown	43	37.72	30	27.78	
ER status					
Negative	47	41.23	69	63.89	**<0.001**
Positive	67	58.77	39	36.11	
PR status					
Negative	72	63.16	88	81.48	**<0.001**
Positive	42	36.84	20	18.52	
Ki‐67					
<20%	12	10.53	2	1.85	**0.011**
≥20%	102	89.47	106	98.15	
Molecular subtype					
HER2+/HR‐	47	41.23	67	62.04	**0.002**
HER2+/HR+	68	59.65	41	37.96	
Neoadjuvant‐targeted therapy					
H	75	65.79	44	40.74	**<0.001**
HP	16	14.04	49	45.37	
H + Pyrotinib	22	19.3	15	13.89	
HL	1	0.88	0	0	
Enough cycle					
Yes	103	90.35	107	99.07	**0.005**
No	11	9.65	1	0.93	
Local therapy					
Breast‐conserving surgery	14	12.28	8	7.41	0.265
Mastectomy	100	87.72	100	92.59	
Axillary surgery					
SLNB	17	14.91	18	16.67	0.854
ALND	97	85.09	90	83.33	

*Note*: The information highlighting statistically significant differences is emphasized in bold.

### 3.2 Tumor mutation burden landscape of Chinese HER2‐positive breast cancer

**FIGURE 1:**
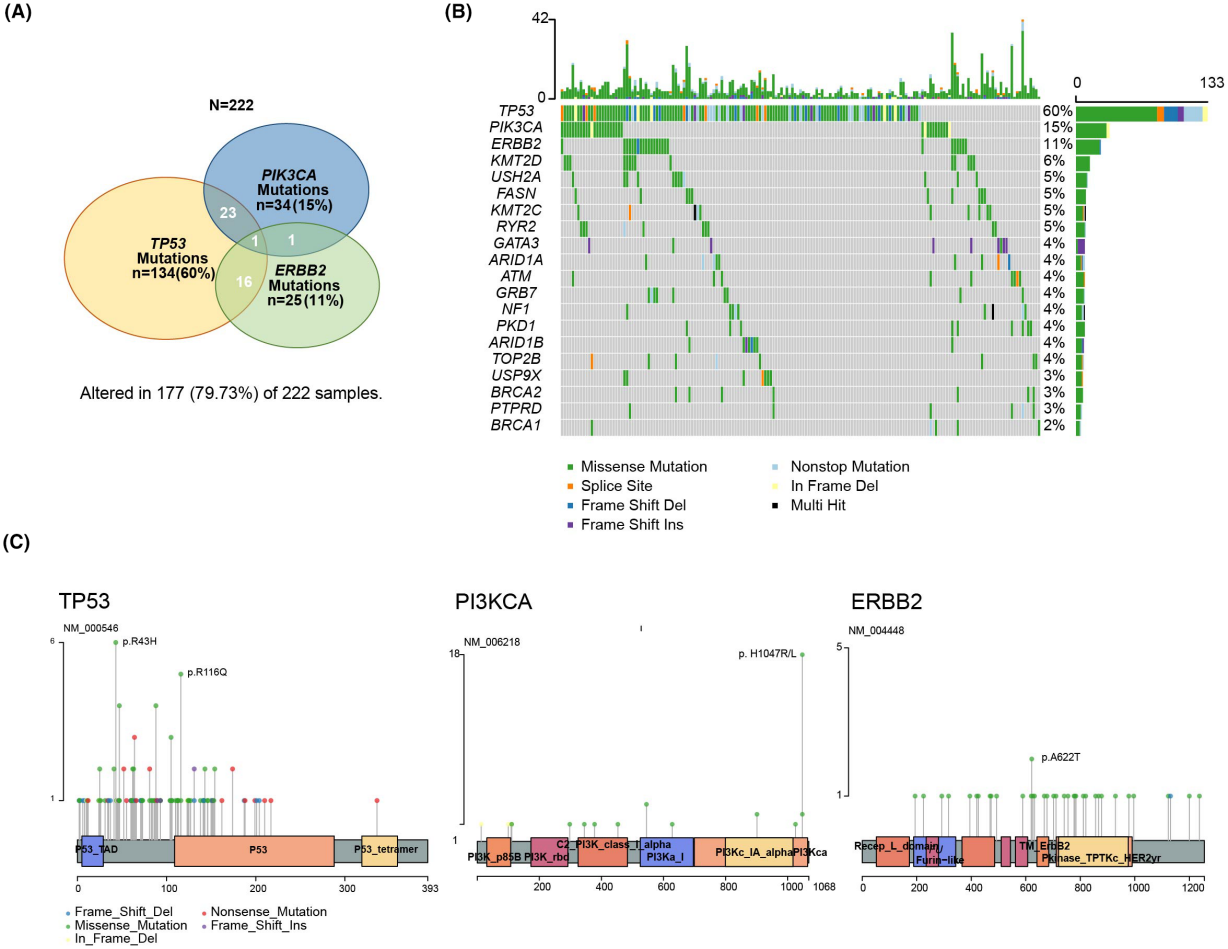
The mutational landscape of 222 Chinese patients with HER2‐positive breast cancer. (A) The number and proportion of the top three mutated genes in the cohort. (B) Waterfall plot summarizing the mutational landscape of the cohort. Each column represents a patient, and each row represents a gene. Numbers on the left represent the percentage of patients with mutations in a specific gene. The top plot represents the overall number of mutations a patient carried. Different colors denote different types of mutations. (C) Lollipop diagrams depicting the type and specific locations of TP53, PIK3CA, and ERBB2 mutations. Colored boxes depict the different functional domains along the gene. Colored circles denote the type of mutation, while the location of the circle specifies the mutation site. The length of the lollipop represents the number of patients harboring a specific variant. The legend on the right side summarizes the total number of mutation types, such as missense, truncating, inframe, and other mutations.

Analysis of somatic mutations in tumor samples demonstrated that the most frequently mutated gene was TP53 (60%), followed by PIK3CA (15%) and ERBB2 (11%) (Figure 1A). We listed the top 20 mutated genes with a waterfall plot in Figure 1B. Genes were altered in 177 (79.73%) of 222 samples in our cohort, and most were missense mutations. Among the mutation sites of TP53, the hotspot mutation sites included p.R43H, p.R116Q, p.Y88C, p.H47R, P.105i, p.N131fs, and p.R64X. The most common mutation sites in PIK3CA were p.H1047R and p.H1047L. However, ERBB2 mutations were scattered and showed no obvious hotspots (Figure 1C).

**FIGURE 2:**
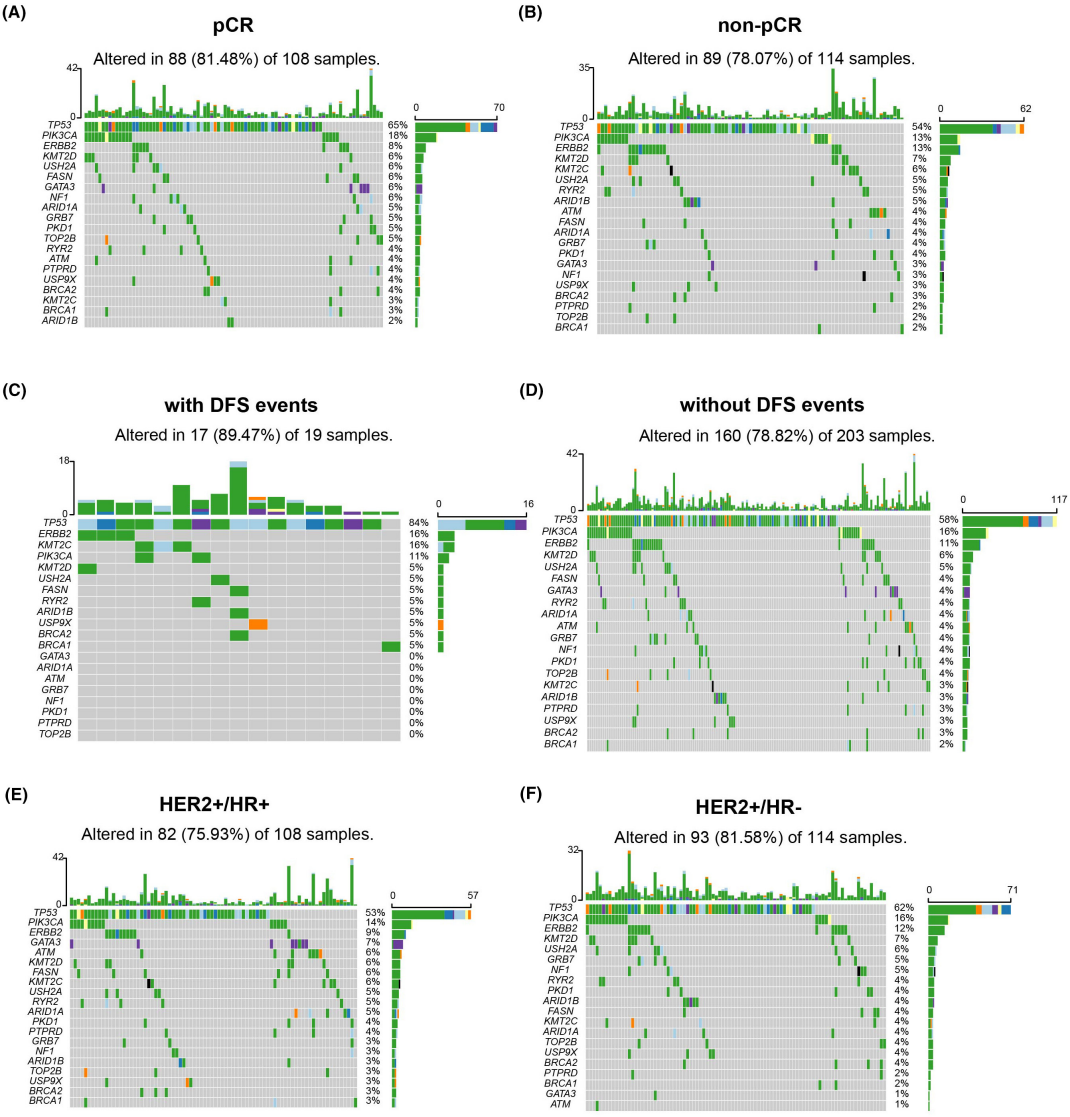
Mutational landscape according to NAT efficacy, DFS events, and HR status. (A, B) Waterfall plot demonstrating the mutational landscape of patients with pCR and non‐pCR. (C, D) Waterfall plot demonstrating the mutational landscape of patients with or without DFS events. (E, F) Waterfall plot demonstrating the mutational landscape of patients with HER2+/HR+ and HER2+/HR‐ status.

We set out to investigate whether somatic mutations influence the efficacy of NAT in HER2‐positive BC. Our investigation revealed that the occurrence of mutation events in patients with pCR and non‐pCR was 88/108 (81.48%) and 89/114 (78.07%), respectively (Figure 2A,B). Genetic mutations between pCR and non‐pCR patients are listed in Table S2, which shows no statistical diversity.

Within our cohort, 19 individuals experienced DFS events during follow‐up period. The most frequently mutated gene was TP53 (84%), followed by ERBB2 (16%) and KMT2C (16%), in patients who experienced DFS events (Figure 2C,D). Upon scrutinizing the mutation profiles, we uncovered that KMT2C (*p =* 0.036) and TP53 (*p =* 0.037) mutations were significantly increased in patients with DFS (Table S3).

### 3.3 Patients' mutational profiles vary depending on HR status

**FIGURE 3:**
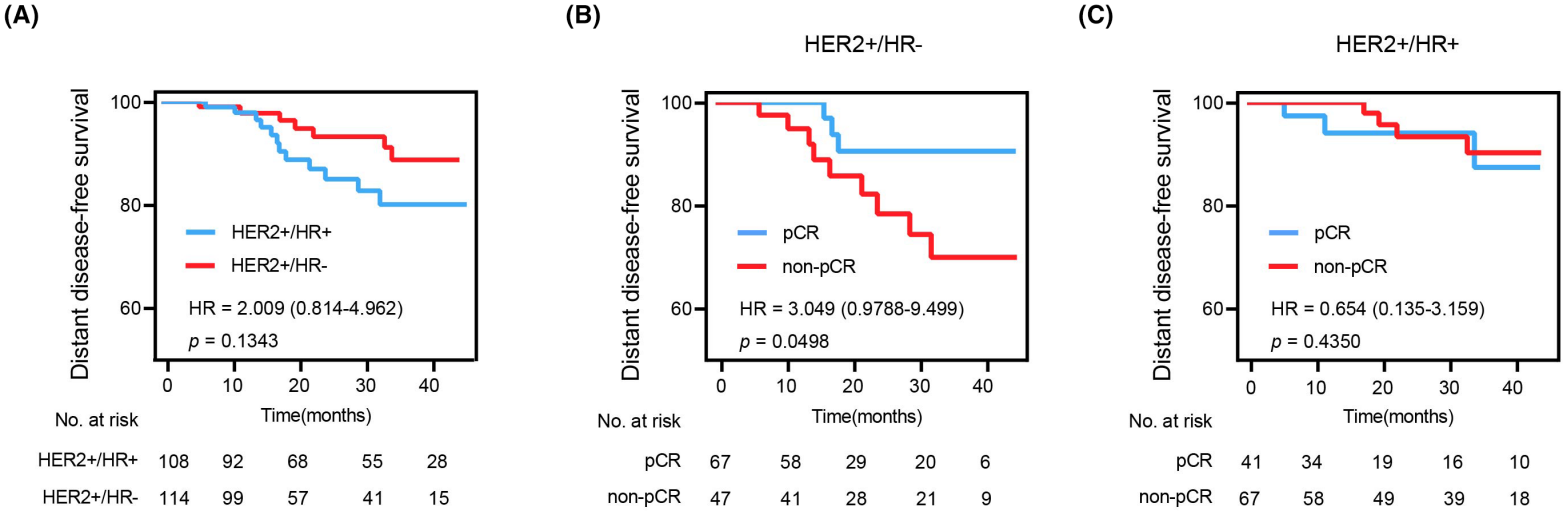
Survival analyses of patients. (A) Survival curve according to HR status. (B) Survival curve according to whether pCR was achieved in HR‐ patients. (C) Survival curve according to whether pCR was achieved in HR+ patients.

The primary end point of NAT is pathological complete response (pCR); the prognosis of patients who achieve pathological complete response (pCR) was observed to be significantly better than that of non‐pCR patients Our analysis also sought to explore the potential impact of HR status on the survival outcomes of HER2‐positive breast cancer patients undergoing NAT. There was no significant difference in DFS between HR+ and HR‐ HER2‐positive BC patients (HR = 2.009, *p =* 0.1343), while the survival curves showed some trends (Figure 3A). However, the effect of pCR on individual survival varies when diverse molecular subtypes are involved. Only in the HER2+/HR‐ group there was a significant difference between pCR and non‐pCR patients (HR = 3.049, *p =* 0.0498) (Figure 3B). Conversely, in the HER2+/HR+ group, there was no statistical significance (HR = 0.654, *p =* 0.4350) (Figure 3C).

In HER2‐positive BC, our results show that the outcomes of patients vary according to HR status among both pCR and non‐pCR patients. A comparison of genetic mutations between HR+ and HR‐ patients is shown in Table S4. We found that in HR+ patients, ATM (*p =* 0.016) and GATA3 (*p =* 0.016) mutations were more frequent (Figure 2E,F).

We then assessed the mutational landscape of pCR and non‐pCR patients with or without HR positivity. The rates of mutation events among the four groups were 75.61% (31/41), 76.12% (51/67), 83.58% (56/67), and 80.85% (38/47) (Figure S1). There was no significant difference in genetic mutations between pCR and non‐pCR patients in the HR+ or HR‐ cohort (Table S5 and S6).

### 3.4 Mutation genotypes associated with clinicopathological features and prognosis

**FIGURE 4:**
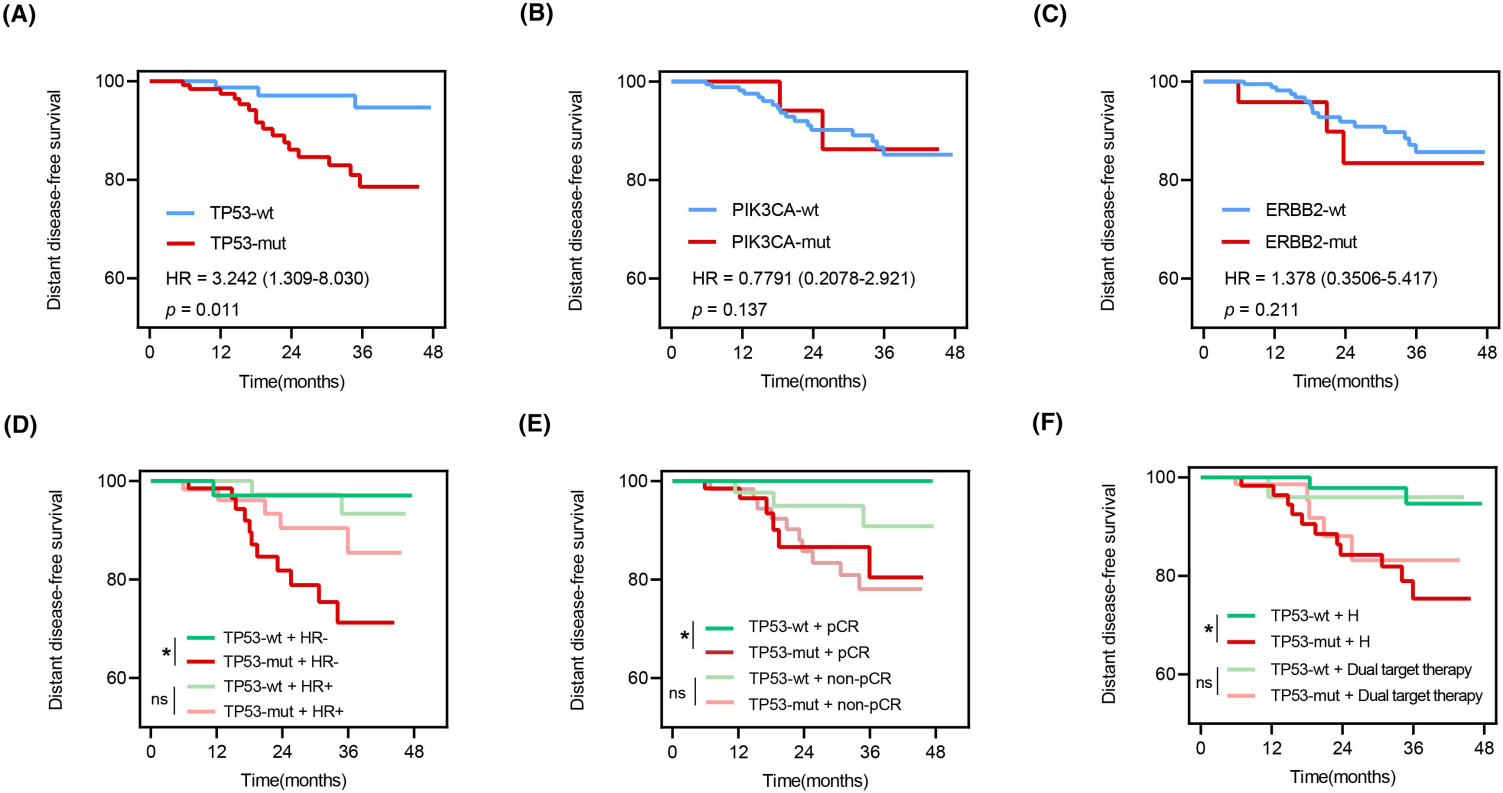
Survival analysis of the top three mutations in patients. (A) Survival curve according to TP53 mutation (B) Survival curve according to PIK3CA mutation (C) Survival curve according to ERBB2 mutation. Survival analysis based on TP53 mutation (D) with or without HR positivity, (E) pCR, and (F) dual‐targeted therapy.

Kaplan–Meier analysis showed significant differences in DFS between the TP53‐mutated and wild‐type groups (HR = 3.242, *p =* 0.011), as shown in Figure 4A. PIK3CA (HR = 0.779, *p =* 0.137) and ERBB2 (HR = 1.378, *p =* 0.211) mutations were not significantly associated with prognosis (Figure 4B,C).

Further analysis of the cohort unveiled noteworthy associations between TP53 mutations and prognosis in the HER2+/HR‐ genotyped cohort (HR = 3.712, *p =* 0.027) but showed no significance in the HER2+/HR+ cohort (HR = 2.284, *p =* 0.276) (Figure 4D). Furthermore, among pCR patients, TP53 status showed more significance in predicting DFS survival (HR = 6.253, *p =* 0.027) than among non‐pCR patients (HR = 2.301, *p =* 0.137) (Figure 4E). TP53 mutations had prognostic significance in patients who received herceptin‐only neoadjuvant‐targeted therapy (HR = 4.145, *p =* 0.011) but not in those who received dual‐targeted NAT (HR = 2.069, *p =* 0.405) (Figure 4F).

Similarly, we investigated the potential impact PIK3CA and ERBB2 mutations on HER2‐positive BC patients with varying clinical characteristics. Figure S2 indicates that neither PI3KCA nor ERBB2 mutations affected patient outcomes, even in the presence of inconsistent HR, neoadjuvant efficacy, and targeted therapy regimens.

## 4 DISCUSSION

As oncology knowledge, key technology and clinical guidelines continue to be updated, molecular profiling has undergone revolutionary changes in the last few years. Treatment decisions in BC rely primarily on tumor molecular subtypes, which depend on the expression of the estrogen receptor, progesterone receptor, and HER2 receptor.^20^ As BC heterogeneity manifests itself, more accurate classification and tumor characterization at the genomic level are still urgently needed.^21^ There have been many studies of genetic mutations in BC over the past decade,^22^ but the mapping of genetic mutations in HER2‐positive BC remains insufficient.^16^ Our research aimed to first depict a molecular plus genomic mutation landscape of Chinese HER2‐positive BC patients undergoing NAT.

It is well recognized that HER2+/HR‐ BC patients benefited more from targeted therapy than HER2+/HR+ patients (OR = 2.34; 95% CI = 1.99 to 2.75).^23^ Analysis of patients with varying treatment responses revealed that those who were ER‐positive and PR‐positive had lower rates of pCR, reason for it may be that HR status promotes activation of the HER2 signaling pathway.^24^ Furthermore, the status of HR has a significant relationship with the prognosis of HER2‐positive patients. In our cohort of HER2‐positive patients undergoing targeted NAT, the presence or absence of pCR showed prognostic differences only in HR‐negative patients, suggesting the long‐term prognostic significance of HR‐receptor status in this subgroup. Consequently, to further explore the genetic mutation profiles of HER2‐positive breast cancer patients, we discuss them separately based on HR‐receptor status.

In our analysis of genetic mutations, HR+ patients had more ATM mutations, and a high expression level of ATM has been reported to be associated with an increased risk of pancancer, including breast cancer, and may lead to chemotherapy resistance.^25^, ^26^ GATA3 mutation is one of the most common mutations in BC,^27^ and GATA3 mutation is associated with a poorer prognosis.^28^ In our cohorts, we found an elevated frequency of GATA3 mutations in HR+ patients compared to HR‐ patients. These changes in the mutational profile may provide insight into the fact that patients with HER2+/HR+ BC have a worse response to HER2‐targeted NAT and a worse prognosis.

Previous studies have identified that the genes with the highest mutation rate in all subtypes of Asian female BC are TP53, PIK3CA, and GATA3.^29^ In our cohort of HER2‐positive BC patients, TP53, PIK3CA, and ERBB2 showed the highest mutation frequency. ERBB2 mutations were reported more frequently in HER2‐positive BC than in other subtypes, which is consistent with our results. According to previous reports, the mutation rates of TP53, PIK3CA, and ERBB2 in all subtypes of BC were 30%–60%,^27^, ^29^ 20%–40%, and 10%–35%, respectively.^30^ We noted that the mutation rate of PIK3CA in our cohort was lower than that in other studies, possibly because we set the VAF > 10% condition to reduce the false positive rate.

As the mutational burden and clonal diversity tend to increase in metastatic BC compared to early‐stage disease,^31^ our findings revealed that patients with recurrent or metastatic events had a higher genetic mutation frequency of 89.47% (17/19). In addition, TP53, KMT2C, and ERBB2 were mutated more frequently in patients with DFS events; among these mutations, both TP53 and KMT2C mutations were found to significantly promote metastatic events. At present, very few studies have been performed on the KMT2C mutation in BC, and the reported results on its role in existing reports are inconsistent.^32^ Recent research has revealed that KMT2C loss led to epithelial‐to‐mesenchymal transition (EMT) in tumors and enhanced metastatic capacity.^33^, ^34^ Therefore, exploring the impact of KMT2C mutations on promoting metastasis in HER2‐positive breast cancer is a topic that deserves further investigation.

We conducted further analysis to explore the correlation between the top three mutated genes and clinicopathological characteristics of HER2‐positive tumors, which demonstrated that TP53 was associated with a poor prognosis. Known as a tumor suppressor gene, TP53 initiates the transcription of genes involved in various cellular processes such as cell cycle arrest, apoptosis, metabolism, DNA repair, and cellular senescence, and mutated TP53 acquires oncogenic properties.^35^ It has been reported that in the neoadjuvant cohort, TP53 mutation was associated with poor response to 5‐fluorouracil, epirubicin, and cyclophosphamide (5‐FEC) (*p =* 0.003).^36^ Conversely, Chen et al. reported that mutant TP53 was associated with increased rates of pCR following NAT.^37^ Notably, none of these studies specifically addressed the relationship between TP53 mutations and prognosis, and sample sizes may have been insufficient.^38^ In our study, we revealed that TP53 mutations did not significantly impact the outcome of NAT in HER2‐positive breast cancer patients. However, interestingly, TP53 mutation affected the prognosis of HER2‐positive BC patients under different conditions. Andersson et al. determined that patients with TP53 mutation BC had worse overall survival (*p* < 0.001), and another study indicated that TP53 mutation in combination with PR negativity was associated with the worst prognosis, which was also consistent with our results.^39^ Our analysis represents the first demonstrate of the significance of TP53 mutations for long‐term DFS in the largest cohort of Chinese HER2+ patients undergoing NAT and focus on the impact of TP53 mutations in the context of HR status, neoadjuvant regimens, and response. Our findings suggest that the mutation status of TP53 holds greater prognostic value for HER2‐positive BC patients with HR negativity, pCR, and herceptin‐only targeted therapy.

Agents targeting TP53 mutations include APR‐246, MK‐1775, ALT‐801, and Kevetrin,^40^ and while these agents are still in early‐stage clinical trials, promising results have been reported in metastatic colon cancer, acute myeloid leukemia, and myelodysplastic syndromes.^41^, ^42^ Targeting TP53 has been proven to improve outcomes among patients with TP53 mutations in HER2+ BC.^43^, ^44^ Our study has brought to the fore the crucial role of addressing TP53 mutations in the treatment of HER2‐positive BC. The clinical challenges posed by TP53 mutations in this context are undeniable; however, the identification of such mutations has also provided a unique opportunity for the development of more personalized and efficacious treatment strategies. The potential benefits of targeting TP53 mutations in the context of HER2‐positive BC are vast and promising, but further research and clinical trials are necessary to fully realize their potential. With continued investigation and innovation, we are hopeful that this avenue of research will lead to significant improvements in the outcomes of patients with HER2‐positive BC. This retrospective study is not without its limitations. To mitigate the potential impact caused by the small probability events, we deliberately placed less emphasis on genes with low mutation frequency so that our results could be obtained with higher robustness and reliability. Throughout the data processing phase, we attempted to unraveling the influence of varying HR receptor statuses on the efficacy of NAT for HER2‐positive BC in terms of genetic mutations. Despite our comprehensive analysis of sample genetic mutations across varying HR statuses, the observed inconsistencies do not provide a definitive explanation regarding the ability of patients in diverse molecular or genetic subgroups to achieve pCR with NAT. Although we also sought to explore the relationship between the response after neoadjuvant C2 and genetic mutations, the significant difference could not be reflected, and an expanded cohort for more comprehensive investigation are needed. (Table S7).

To the best of our knowledge, this is the most extensive investigation to date addressing the relevance of diverse genotype mutations in Chinese HER2‐positive BC patients undergoing NAT with DFS. Our study uniquely sheds light on the impact of TP53 mutation on HR status, neoadjuvant regimen, and efficacy, thus providing a valuable reference for BC precision therapy. In summary, our work underscored the critical importance of a more precise formulation of molecular and genetic mutation subtypes in BC patients to enable more personalized and efficacious treatment strategies.

## 5 CONCLUSION

The achievement of pCR plays a pivotal role in determining the prognosis of patients with HER2‐positive BC undergoing NAT. Furthermore, the prognostic impact of pCR is particularly significant in the cohort of patients who are HR‐negative rather than HR‐positive, which suggests that HR‐ patients may derive greater benefits from NAT, suggesting a molecular‐level significance.

Furthermore, this study provides a comprehensive elucidation of the mutational profiles in patients with neoadjuvant HER2‐positive BC. The frequency of somatic alterations in TP53, PIK3CA, and ERBB2 was highest in Chinese women with HER2‐positive BC. Somatic alterations in TP53 proved to be a crucial determinant of prognosis in HER2+/HR‐ BC patients, further highlighting the clinical significance of this genetic mutation. Importantly, the impact of TP53 mutation on patient prognosis displayed different across variability targeted therapy and neoadjuvant efficacies. Further studies delving into these genetic mutations and their underlying mechanisms hold immense potential in delineating biological susceptibilities in HER2+ BC patients, ultimately leading to enhanced treatment options.

## AUTHOR CONTRIBUTIONS

**Min Xiong:** Conceptualization (equal); data curation (equal); formal analysis (equal); writing – original draft (equal). **Xuliren Wang:** Data curation (equal); formal analysis (equal); project administration (equal); writing – original draft (equal). **Douwaner Liu:** Data curation (equal); formal analysis (equal). **Bingqiu Xiu:** Methodology (equal). **Qi Zhang:** Methodology (equal). **Weiru Chi:** Data curation (equal). **Chihwan Goh:** Data curation (equal). **Liyi Zhang:** Formal analysis (equal). **Ming Chen:** Investigation (equal). **Hengyu Ren:** Formal analysis (equal). **Zhiming Shao:** Resources (equal). **Ben‐long Yang:** Methodology (equal); project administration (equal); writing – review and editing (equal). **Jiong Wu:** Funding acquisition (equal); methodology (equal); project administration (equal); writing – review and editing (equal).

## FUNDING INFORMATION

This work was supported by grants from the National Natural Science Foundation of China (82072919).

## CONFLICT OF INTEREST STATEMENT

The authors declare that they have no competing interests.

## ETHICS APPROVAL AND CONSENT TO PARTICIPATE

This research gained approval from the Medical Ethics Committee of Fudan University Shanghai Cancer Center.

## CONSENT FOR PUBLICATION

All authors agreed on this article.

## Supporting information


Figure S1.

Figure S2.

Table S1.

Table S2.

Table S3.

Table S4.

Table S5.

Table S6.

Table S7.

## Data Availability

The data that support the findings of this study are available from the corresponding author upon request.
