# Supplementary figures and images for: Somatic mutations in a multigene panel and impact on prognosis based on TP53 status in Chinese HER2‐positive patients undergoing neoadjuvant therapy: A single‐institution retrospective cohort
Source: Cancer Med. 2024 Feb 1;13(2):e6955. doi: 10.1002/cam4.6955 (PMC10832311; doi:10.1002/cam4.6955)

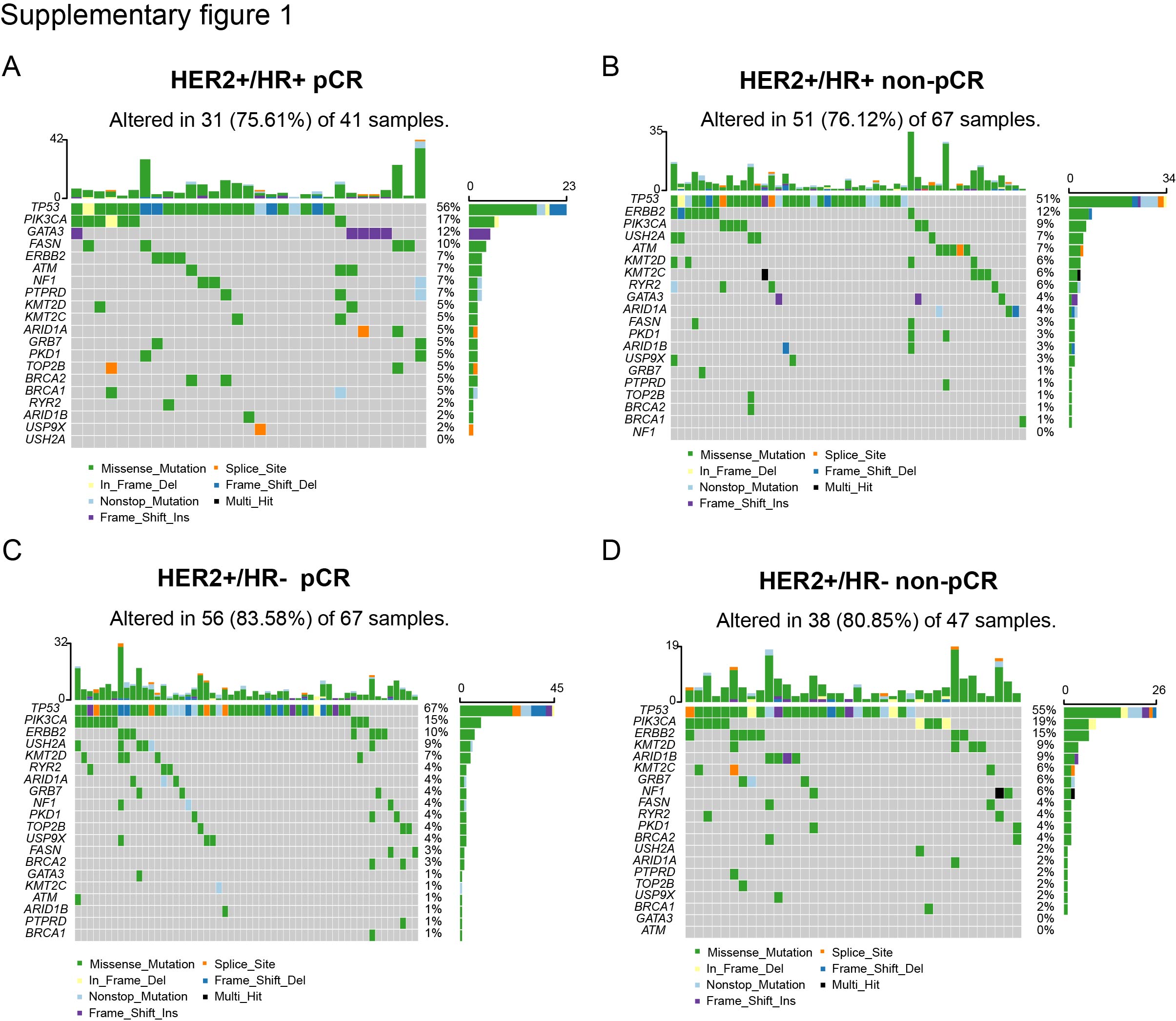

Supplement: Supplementary file 1 — Figure S1. [file CAM4-13-e6955-s004.jpg]

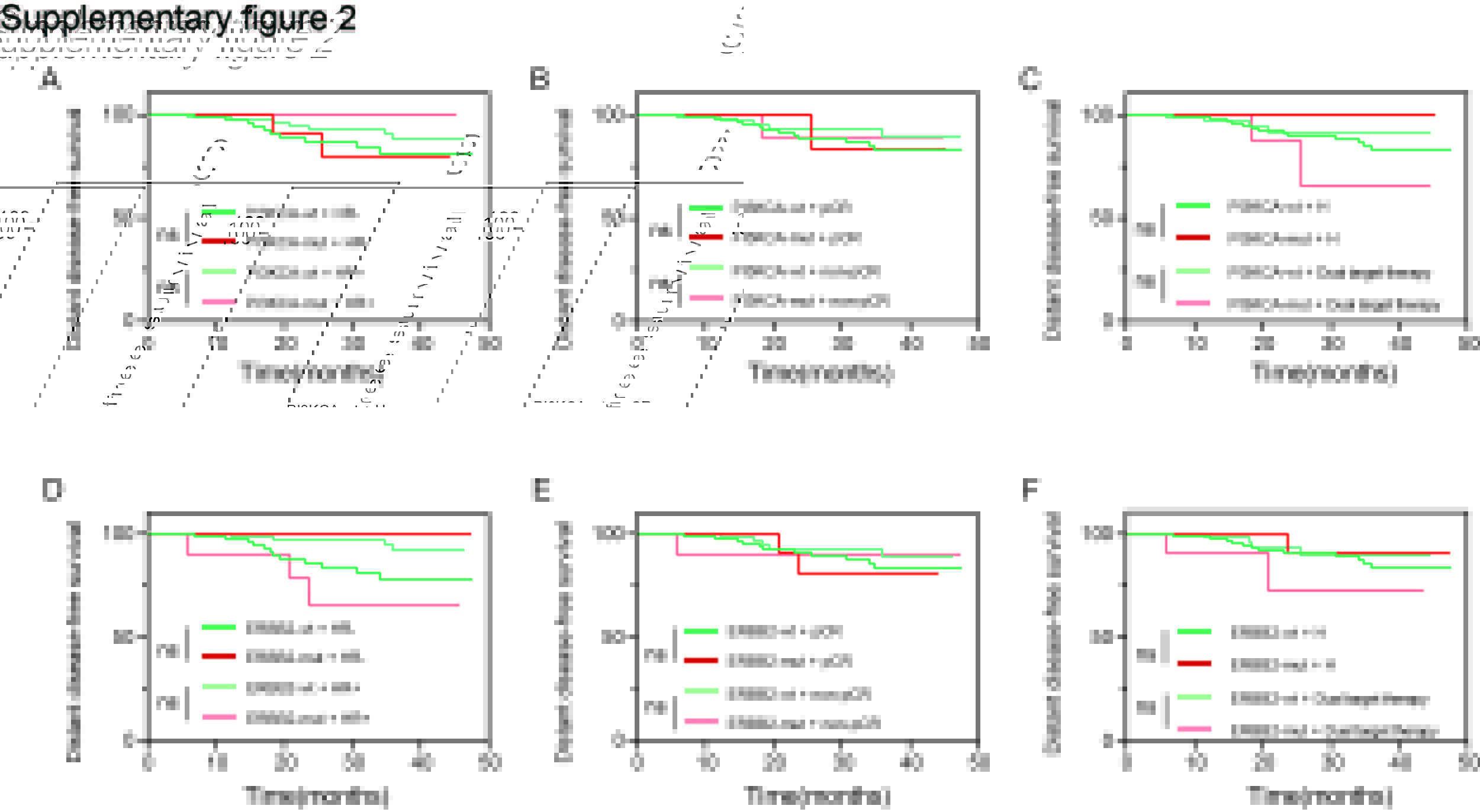

Supplement: Supplementary file 2 — Figure S2. [file CAM4-13-e6955-s005.jpg]
