# Supplementary material for: Somatic mutations in a multigene panel and impact on prognosis based on TP53 status in Chinese HER2‐positive patients undergoing neoadjuvant therapy: A single‐institution retrospective cohort
Source: Cancer Med. 2024 Feb 1;13(2):e6955. doi: 10.1002/cam4.6955 (PMC10832311; doi:10.1002/cam4.6955)
Supplement: Supplementary file 3 — Table S1. [file CAM4-13-e6955-s002.docx]

Supplementary table 1 DFS events during the follow-up

| Recurrence events | Number | Incidence (%) |
| --- | --- | --- |
| Metastatic events | 19 | 8.56 |
| Brain | 7 | 3.15 |
| Liver | 6 | 2.70 |
| Lung | 4 | 1.80 |
| Bone | 4 | 1.80 |
| Lymph node | 5 | 2.25 |
| Chest wall | 1 | 0.45 |
