# Supplementary material for: Somatic mutations in a multigene panel and impact on prognosis based on TP53 status in Chinese HER2‐positive patients undergoing neoadjuvant therapy: A single‐institution retrospective cohort
Source: Cancer Med. 2024 Feb 1;13(2):e6955. doi: 10.1002/cam4.6955 (PMC10832311; doi:10.1002/cam4.6955)
Supplement: Supplementary file 4 — Table S2. [file CAM4-13-e6955-s008.docx]

Supplementary Table 2 Genetic mutations between pCR and non-pCR patients

| Gene | pCR(n=108) | | non-pCR(n=114) | | *p* |
| --- | --- | --- | --- | --- | --- |
|  | WT | Amplified/Mutated | WT | Amplified/Mutated |  |
| **Mutation genes** |  |  |  |  |  |
| ARID1A | 103 | 5 | 110 | 4 | 0.743 |
| ARID1B | 105 | 3 | 108 | 6 | 0.500 |
| ATM | 104 | 4 | 109 | 5 | 0.797 |
| BRCA1 | 105 | 3 | 112 | 2 | 0.611 |
| BRCA2 | 104 | 4 | 111 | 3 | 0.649 |
| ERBB2 | 98 | 10 | 99 | 15 | 0.401 |
| FASN | 102 | 6 | 110 | 4 | 0.530 |
| GATA3 | 102 | 6 | 111 | 3 | 0.322 |
| GRB7 | 103 | 5 | 110 | 4 | 0.743 |
| KMT2C | 105 | 3 | 106 | 8 | 0.217 |
| KMT2D | 101 | 7 | 106 | 8 | 0.874 |
| NF1 | 102 | 6 | 110 | 4 | 0.530 |
| PIK3CA | 89 | 19 | 99 | 15 | 0.456 |
| PKD1 | 103 | 5 | 110 | 4 | 0.743 |
| PTPRD | 104 | 4 | 112 | 2 | 0.436 |
| RYR2 | 104 | 4 | 108 | 6 | 0.749 |
| TOP2B | 103 | 5 | 111 | 3 | 0.490 |
| TP53 | 38 | 70 | 50 | 64 | 0.217 |
| USH2A | 102 | 6 | 108 | 6 | 0.923 |
| USP9X | 104 | 4 | 111 | 3 | 0.649 |
