# Supplementary material for: Somatic mutations in a multigene panel and impact on prognosis based on TP53 status in Chinese HER2‐positive patients undergoing neoadjuvant therapy: A single‐institution retrospective cohort
Source: Cancer Med. 2024 Feb 1;13(2):e6955. doi: 10.1002/cam4.6955 (PMC10832311; doi:10.1002/cam4.6955)
Supplement: Supplementary file 5 — Table S3. [file CAM4-13-e6955-s009.docx]

Supplementary table 3

Genetic mutations between patients with or without DFS events

| Mutation genes | With DFS events (N=19) | | Without DFS events（N=203） | | *p* |
| --- | --- | --- | --- | --- | --- |
|  | WT | Amplified/Mutated | WT | Amplified/Mutated |  |
| ARID1A | 19 | 0 | 194 | 9 | 0.661 |
| ARID1B | 18 | 1 | 195 | 8 | 0.781 |
| ATM | 19 | 0 | 194 | 9 | 0.661 |
| BRCA1 | 18 | 1 | 199 | 4 | 0.375 |
| BRCA2 | 18 | 1 | 197 | 6 | 0.587 |
| ERBB2 | 16 | 3 | 181 | 22 | 0.517 |
| FASN | 18 | 1 | 194 | 9 | 0.868 |
| GATA3 | 19 | 0 | 194 | 9 | 0.661 |
| GRB7 | 19 | 0 | 194 | 9 | 0.661 |
| KMT2C | 16 | 3 | 195 | 8 | **0.036** |
| KMT2D | 18 | 1 | 189 | 14 | 0.787 |
| NF1 | 19 | 0 | 193 | 10 | 0609 |
| PIK3CA | 17 | 2 | 171 | 32 | 0.548 |
| PKD1 | 19 | 0 | 194 | 9 | 0.661 |
| PTPRD | 19 | 0 | 197 | 6 | 0.867 |
| RYR2 | 18 | 1 | 194 | 9 | 0.868 |
| TOP2B | 19 | 0 | 195 | 8 | 0.720 |
| TP53 | 3 | 16 | 85 | 118 | **0.037** |
| USH2A | 18 | 1 | 192 | 11 | 0.977 |
| USP9X | 19 | 0 | 196 | 7 | 0.788 |
