# Supplementary material for: Somatic mutations in a multigene panel and impact on prognosis based on TP53 status in Chinese HER2‐positive patients undergoing neoadjuvant therapy: A single‐institution retrospective cohort
Source: Cancer Med. 2024 Feb 1;13(2):e6955. doi: 10.1002/cam4.6955 (PMC10832311; doi:10.1002/cam4.6955)
Supplement: Supplementary file 6 — Table S4. [file CAM4-13-e6955-s007.docx]

Supplementary table 4

Genetic mutations between HR+ and HR- patients

| Mutation genes | HR+(N=108) | | HR-(N=114) | | *p* |
| --- | --- | --- | --- | --- | --- |
|  | WT | Amplified/Mutated | WT | Amplified/Mutated |  |
| ARID1A | 103 | 5 | 110 | 4 | 0.743 |
| ARID1B | 104 | 4 | 109 | 5 | 0.797 |
| ATM | 100 | 8 | 113 | 1 | **0.016** |
| BRCA1 | 105 | 3 | 112 | 2 | 0.611 |
| BRCA2 | 105 | 3 | 110 | 4 | 0.756 |
| ERBB2 | 97 | 11 | 100 | 14 | 0.675 |
| FASN | 102 | 6 | 110 | 4 | 0.530 |
| GATA3 | 100 | 8 | 113 | 1 | **0.016** |
| GRB7 | 105 | 3 | 108 | 6 | 0.500 |
| KMT2C | 101 | 7 | 110 | 4 | 0.365 |
| KMT2D | 102 | 6 | 105 | 9 | 0.596 |
| NF1 | 105 | 3 | 107 | 7 | 0.334 |
| PIK3CA | 93 | 15 | 95 | 19 | 0.582 |
| PKD1 | 104 | 4 | 109 | 5 | 0.797 |
| PTPRD | 104 | 4 | 112 | 2 | 0.436 |
| RYR2 | 103 | 5 | 109 | 5 | 0.930 |
| TOP2B | 104 | 4 | 110 | 4 | 0.938 |
| TP53 | 49 | 59 | 39 | 75 | 0.101 |
| USH2A | 103 | 5 | 107 | 7 | 0.769 |
| USP9X | 105 | 3 | 110 | 4 | 0.756 |
