# Supplementary material for: Somatic mutations in a multigene panel and impact on prognosis based on TP53 status in Chinese HER2‐positive patients undergoing neoadjuvant therapy: A single‐institution retrospective cohort
Source: Cancer Med. 2024 Feb 1;13(2):e6955. doi: 10.1002/cam4.6955 (PMC10832311; doi:10.1002/cam4.6955)
Supplement: Supplementary file 7 — Table S5. [file CAM4-13-e6955-s006.docx]

Supplementary table 5

Genetic mutations between pCR and non-pCR in HR+ patients

| Mutation genes | HR+ pCR (N=41) | | HR+ non-pCR（N=67） | | *p* |
| --- | --- | --- | --- | --- | --- |
|  | WT | Amplified/Mutated | WT | Amplified/Mutated |  |
| ARID1A | 42 | 2 | 61 | 3 | 0.973 |
| ARID1B | 42 | 2 | 62 | 2 | 0.703 |
| ATM | 41 | 3 | 59 | 5 | 0.846 |
| BRCA1 | 42 | 2 | 63 | 1 | 0.376 |
| BRCA2 | 42 | 2 | 63 | 1 | 0.376 |
| ERBB2 | 41 | 3 | 56 | 8 | 0.520 |
| FASN | 40 | 4 | 62 | 2 | 0.222 |
| GATA3 | 39 | 5 | 61 | 3 | 0.267 |
| GRB7 | 42 | 2 | 63 | 1 | 0.376 |
| KMT2C | 42 | 2 | 59 | 5 | 0.698 |
| KMT2D | 42 | 2 | 60 | 4 | 0.705 |
| NF1 | 41 | 3 | 64 | 0 | 0.118 |
| PIK3CA | 35 | 9 | 58 | 6 | 0.115 |
| PKD1 | 42 | 2 | 62 | 2 | 0.703 |
| PTPRD | 41 | 3 | 63 | 1 | 0.302 |
| RYR2 | 43 | 1 | 60 | 4 | 0.646 |
| TOP2B | 42 | 2 | 62 | 2 | 0.703 |
| TP53 | 21 | 23 | 28 | 36 | 0.699 |
| USH2A | 44 | 0 | 59 | 5 | 0.157 |
| USP9X | 43 | 1 | 62 | 2 | 0.792 |
