# Supplementary material for: Somatic mutations in a multigene panel and impact on prognosis based on TP53 status in Chinese HER2‐positive patients undergoing neoadjuvant therapy: A single‐institution retrospective cohort
Source: Cancer Med. 2024 Feb 1;13(2):e6955. doi: 10.1002/cam4.6955 (PMC10832311; doi:10.1002/cam4.6955)
Supplement: Supplementary file 8 — Table S6. [file CAM4-13-e6955-s001.docx]

Supplementary table 6

Genetic mutations between pCR and non-pCR in HR- patients

| Gene | HR- pCR (N=67) | | HR- non-pCR（N=47） | | *p* |
| --- | --- | --- | --- | --- | --- |
|  | WT | Amplified/Mutated | WT | Amplified/Mutated |  |
| **Mutation genes** |  |  |  |  |  |
| ARID1A | 64 | 3 | 46 | 1 | 0.642 |
| ARID1B | 66 | 1 | 43 | 4 | 0.158 |
| ATM | 66 | 1 | 47 | 0 | 0.643 |
| BRCA1 | 66 | 1 | 46 | 1 | 0.800 |
| BRCA2 | 65 | 2 | 45 | 2 | 0.718 |
| ERBB2 | 60 | 7 | 40 | 7 | 0.566 |
| FASN | 65 | 2 | 45 | 2 | 0.718 |
| GATA3 | 66 | 1 | 47 | 0 | 0.643 |
| GRB7 | 64 | 3 | 44 | 3 | 0.689 |
| KMT2C | 66 | 1 | 44 | 3 | 0.304 |
| KMT2D | 62 | 5 | 43 | 4 | 0.838 |
| NF1 | 64 | 3 | 43 | 4 | 0.444 |
| PIK3CA | 57 | 10 | 38 | 9 | 0.614 |
| PKD1 | 64 | 3 | 45 | 2 | 0.886 |
| PTPRD | 66 | 1 | 46 | 1 | 0.19 |
| RYR2 | 64 | 3 | 45 | 2 | 0.955 |
| TOP2B | 64 | 3 | 46 | 1 | 0.512 |
| TP53 | 20 | 47 | 19 | 28 | 0.316 |
| USH2A | 61 | 6 | 46 | 1 | 0.237 |
| USP9X | 64 | 3 | 46 | 1 | 0.642 |
