# Supplementary material for: Somatic mutations in a multigene panel and impact on prognosis based on TP53 status in Chinese HER2‐positive patients undergoing neoadjuvant therapy: A single‐institution retrospective cohort
Source: Cancer Med. 2024 Feb 1;13(2):e6955. doi: 10.1002/cam4.6955 (PMC10832311; doi:10.1002/cam4.6955)
Supplement: Supplementary file 9 — Table S7. [file CAM4-13-e6955-s003.docx]

Supplementary table 7

The relationship between tumor neoadjuvant efficacy and genetic mutations evaluated after C2

| Status after C2 | PR (N=134) | | SD（N=14） | | *p* |
| --- | --- | --- | --- | --- | --- |
|  | WT | Mutated | WT | Mutated |  |
| TP53 | 53 | 81 | 4 | 10 | 0.425 |
| PI3KCA | 54 | 20 | 12 | 2 | 0.323 |
| ERBB2 | 62 | 12 | 12 | 2 | 0.856 |
